# Nutritional composition and acceptability of biscuits fortified with palm weevil larvae (*Rhynchophorus phoenicis Fabricius)* and orange‐fleshed sweet potato among pregnant women

**DOI:** 10.1002/fsn3.1024

**Published:** 2019-04-22

**Authors:** Jessica Ayensu, Herman Lutterodt, Reginald Adjetey Annan, Anthony Edusei, Su Peng Loh

**Affiliations:** ^1^ Department of Biochemistry and Biotechnology Kwame Nkrumah University of Science and Technology Kumasi Ghana; ^2^ Department of Clinical Nutrition and Dietetics University of Cape Coast Cape Coast Ghana; ^3^ Department of Food Science and Technology Kwame Nkrumah University of Science and Technology Kumasi Ghana; ^4^ School of Public Health Kwame Nkrumah University of Science and Technology Kumasi Ghana; ^5^ Department of Nutrition and Dietetics Universiti Putra Serdang Malaysia

**Keywords:** fortified biscuits, nutrition, orange‐fleshed sweet potato, pregnant women, rats, *Rhynchophorus phoenicis Fabricius*

## Abstract

Edible insects are currently being promoted as an inexpensive alternative source of protein in underdeveloped countries due to the rising cost of conventional animal protein and the foreseen future deficit in its supply. A supplemental palm weevil larvae and orange‐fleshed sweet potato biscuit was developed as part of efforts to understand the nutritional benefits of edible insects and to predict whether these benefits will contribute to better nutrition among pregnant women in Ghana. The palm weevil larvae flour and the orange‐fleshed sweet potato flour were mixed with wheat flour in three formulations that had 0, 35, and 70% of palm weevil larvae flour, before being made into biscuits. The biscuits were subjected to proximate and mineral content analysis and sensory evaluation. Proximate and mineral composition of the biscuits increased with increasing levels of palm weevil larvae flour substitution. Among the blends, biscuits containing 70% palm weevil larvae had the highest energy and fat content, and protein content also increased by 45% compared with biscuits made from 100% wheat flour. Calcium, iron, and zinc levels also increased with increasing levels of palm weevil larvae flour substitution. However, carbohydrate and crude fiber concentrations of the biscuits decreased with increasing substitution. The overall acceptability of the biscuits as determined by sensory evaluation using pregnant women was high. Biscuits fortified with palm weevil larvae can be a nutritious snack for pregnant women.

## 1 INTRODUCTION

Pregnancy is a crucial period during which good maternal nutrition cannot be neglected. In recent times, it has been found that the nutrition within the first 1,000 days from conception is critical because whatever is lost during the first 1,000 days is usually irreplaceable. It is therefore imperative that pregnant women get the best nutrition in whatever form it is available.

Pregnant women in sub‐Saharan Africa are at risk of poor nutritional status and adverse outcomes due to poverty, suboptimal healthcare facilities, and infections. Food insecurity also contributes to the risk of poor nutrition among pregnant women in sub‐Saharan Africa. Studies in Ghana have shown a high prevalence of nutrient deficiencies such as iron, protein, vitamin A, and folate, which is largely due to the unavailability of adequate food sources (Ayensu, Edusei, Oduro, & Larbie, 2017; Saaka, Oladele, Larbi, & Hoeschle‐Zeledon, 2017). The effects of poor nutrition among a population cannot be underrated. Documented consequences of maternal protein deficiency include anemia, embryonic losses, intrauterine growth restriction, and reduced postnatal growth. Adequate intake of essential nutrients is therefore necessary for averting undesirable birth outcomes. It would also be important to make the food sources of essential nutrients available in a convenient, cheap, and sustainable manner to enable the populace have easy access to them.

Animals are known to be a rich source of protein, and livestock production has over the years been a major source of providing the human species with their protein requirements. In recent times, however, there have been concerns with regard to the environmental effects of livestock production. Increasing animal protein production through the traditional livestock rearing is said to lead to an unsustainable rise in greenhouse emissions (Gerber et al., 2013). Decreasing livestock production on the other hand may be beneficial to the environment but will increase malnutrition incidences, because about 10.7% (815 million) of the world's population currently suffer from chronic undernourishment (FAO, IFAD, UNICEF, WFP, & WHO, 2017). This prompts the need for more balanced and environmentally friendly methods for producing nutritious foods. The use of insects as food is among the many approaches that have been thought to provide a long‐lasting solution to this foreseen future deficit in animal protein supply. Other underutilized indigenous crops such as the orange‐fleshed sweet potato could also provide other nutrients resulting in a balanced diet that pregnant women could rely on to supply their nutritional needs.

The practice of using insects for food although may be new to some populations, especially in Western countries, has existed for thousands of years and is currently practiced by over 2 billion people in 113 countries in Africa, Asia, and Latin America. Beetles, caterpillars, wasps, bees, crickets, grasshoppers, termites, and locusts are common among the over 2000 species of insects known to be consumed by humans (Van Huis et al., 2013). Insects are highly nutritious and rich sources of essential amino acids just as other forms of protein such as beef, fish, and poultry (Payne, Scarborough, Rayner, & Nonaka, 2016; Rumpold & Schlüter, 2015). For instance, consuming 100 g of caterpillars can provide 76% of the recommended daily protein requirements for adults (Agbidye, Ofuya, & Akindele, 2009). In addition to their nutritional benefits, insect production can be advantageous to the environment due to reduced pesticide use and the use of smaller land areas to produce the same quantities of the protein resulting in fewer greenhouse gas emissions and higher efficiency in feed conversion (Oonincx & De Boer, 2012; Ribeiro, Cunha, Sousa‐Pinto, & Fonseca, 2018). Unfortunately, most of these benefits are largely unknown to the public leading to the marginalization of entomophagy even in countries where insects are part of traditional cuisines.

Entomophagy is widespread in Ghana with different insect species being consumed in different regions in the country. The insects commonly consumed in Ghana are termites, shea tree caterpillar, grasshoppers, locust, field cricket, palm weevil larvae and scarab beetle (Anankware, Fening, Osekre, & Obeng‐Ofori, 2015). Among these, the larvae of the African palm weevil (*Rhynchophorus phoenicis Fabricius*), locally known as “Akokono” among the Akans, are the most consumed. The larvae are available all year round in palm‐growing communities but most abundant during the main rainy season (May to October) (Anankware et al., 2015). They are usually roasted, stewed or boiled, and served during storytelling or for special dishes on special occasions. Because of their high protein content, consumption of the larvae is currently being promoted as an alternative source of protein for preventing severe acute malnutrition (SAM) and possibly anemia (Elemo, Elemo, Makinde, & Erukainure, 2011; Parker et al., 2017).

Orange‐fleshed sweet potato (OFSP) is an excellent source of vitamin A and a cheaper source of this vitamin for poor rural and urban families in sub‐Saharan Africa. It is also rich in energy, vitamin B vitamin, vitamins C and K as well as potassium and phosphorus. Research evidence suggests that daily addition of 100 g of OFSP to the diet can prevent vitamin A deficiency (VAD) in children and reduce maternal mortality. One small boiled root of most varieties of OFSP is known to provide 100% of the daily vitamin A recommendations for children and one medium root provides all of the vitamin A needs for most women of reproductive age (Mitra, 2012). Presently, OFSP is underutilized and rarely consumed in most regions of Ghana probably due to low publicity.

Despite the enormous nutritional benefits of eating insects and other indigenous food crops, Westernization, in recent times, has affected the attitudes of people negatively regarding their consumption. It will therefore be prudent to adopt the approach of merging the nutrient‐rich traditional cuisines with popular foods such breads, biscuits, chocolates, and even juices. Biscuits are popular ready‐to‐eat convenient and affordable snacks that are widely consumed by all age groups in many countries and have been used for nutrient supplementation in many cases. The principal ingredients for making them are wheat flour, shortening, and sugar. These ingredients result in products that are high in carbohydrate but low protein and micronutrient contents. We therefore developed a supplemental palm weevil larvae and orange‐fleshed sweet potato biscuit as a snack with the aim of harnessing of the nutritional and health benefits of the palm weevil larvae and orange‐fleshed sweet potato to help improve the protein and vitamin A intakes of pregnant women in Ghana.

## 2 MATERIALS AND METHODS

Three biscuits, of varying palm weevil larvae composition, made with wheat flour were developed based on the current protein dietary reference intake of 71 g/day or 1.1 g/kg body weight/day for pregnant women. It was estimated that pregnant women would obtain at least 70% of their protein needs from household food sources and the palm weevil larvae and orange‐fleshed sweet potato‐enriched biscuits would provide 30% of the remaining needs, whereas the plain wheat biscuits would provide minimal protein. Farmed palm weevil larvae were obtained from the ASPIRE Food Group breeding site, Kwame Nkrumah University of Science and Technology, Kumasi, Ghana. The orange‐fleshed sweet potato flour and other ingredients were purchased from a local market in Kumasi, Ghana. All reagents used in the analysis were of analytical grade, obtained from Chemolab Supplies, Malaysia. The experiment was carried out in the Food Processing Laboratory, Department of Food Science and Technology, Kwame Nkrumah University of Science and Technology (KNUST), Kumasi, Ghana, and the Nutrition Laboratory of the Department of Nutrition and Dietetics, Universiti Putra Malaysia, Malaysia.

### 2.1 Production of palm weevil larvae (*Rhynchophorus phoenicis* Fabricius) and orange‐fleshed sweet potato biscuits

**Figure 1:**
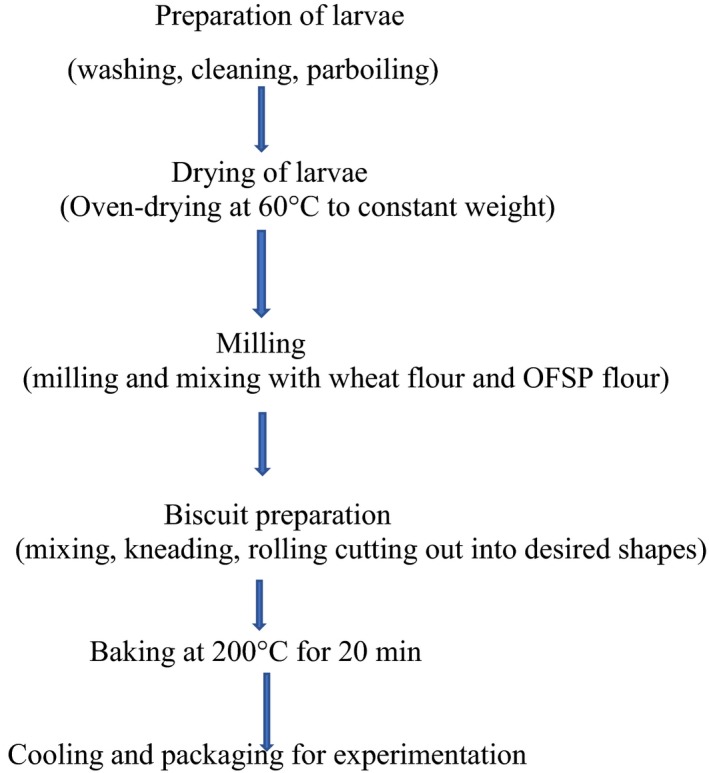
Flowchart for preparation of biscuits enriched with PWL and OFSP

The processes involved in the production of the biscuits are presented in Figure 1. The palm weevil larvae flour (PWLF) and the orange‐fleshed sweet potato flour (OFSPF) were mixed with wheat flour in three formulations that had 0%, 35%, and 70% of the larvae before being made into biscuits. The preparation was done using the creaming method described by Okaka (1997) with slight modifications. The basic formulations were OFSPF, PWLF, and wheat composite flour, fat (30%), sugar (20%), salt (2%), and baking powder (1%). The sugar and fat were mixed to produce a creamy mixture after which the flour and other dry ingredients were weighed out and added. The mixture was extensively blended to form a hard consistent dough. The dough obtained was kneaded for about 5 min, thinly rolled on a wooden board with a rolling pin until a consistent thickness, and cut out (using a biscuit cutter) to desired shapes of similar sizes. The cutout biscuit dough pieces were then arranged on greased baking trays and placed in an oven to bake at 200°C for 20 min. The biscuits were cooled immediately after baking and packed in polyethylene bag. These were sealed and kept at room temperature until used for chemical analysis and sensory evaluation. The biscuits used as the control were produced using wheat flour (100%) following the same procedure.

### 2.2 Proximate analysis

The proximate was carried out according to AOAC (1990), and all analysis was carried out in triplicate.

#### 2.2.1 Determination of moisture content

The moisture content of the formulated biscuits was determined according to the official methods of analysis (AOAC, 1990). Briefly, 2.00 g of each sample was placed into already dried and weighed moisture dishes and dried in an oven (Memmert GmbH) at 105°C for 6 hr, after which the dishes were placed in a desiccator to cool to room temperature, weighed, and placed in the oven and dried overnight till a constant weight was obtained. The moisture content was expressed as a percentage of the average weight loss after drying the sample according to the formula:

$$\frac{W_{2}-W_{3}}{W_{2}-W_{1}}\times 100$$

where *W*_1_ is the weight of the dish; *W*_2_ is the weight of dish and wet sample; and *W*_3_ is the weight of dish and dried sample.

#### 2.2.2 Determination of ash content

The ash of a foodstuff is the inorganic residue left after the organic residue has been burnt away. In this study, the total ash content of the biscuits was determined by incinerating samples overnight in a muffle furnace according to the AOAC, 1990 method. Briefly, 2 g of each sample was transferred into dry and weighed crucibles. The sample in the crucible was charred for 3 hr in an oven and incinerated overnight in a muffle furnace (Thermolyne/Thermo Scientific) at 550°C. Samples were then allowed to cool to room temperature in a desiccator and weighed afterward. The difference in weight was expressed as the percentage total ash of the biscuit samples.

The percentage of fat was calculated according to the formula below:

$$\frac{C-A}{B-A}\times 100$$

where *A* is the weight of the crucible; *B* is the weight of crucible and raw sample; and *C* is the wight of crucible and dried sample.

#### 2.2.3 Determination of crude protein

The Kjeldahl method (AOAC, 1990) was used for determining the crude protein content of biscuits in this study. Briefly, 1 g each of homogenized sample was weighed and transferred into digestion tubes and 0.5 g of Kjeldahl tabs was added to each sample. After this, 12 ml of H_2_SO_4_ was added and the tubes were agitated to wet the samples. The sample was digested at 420°C for an hour when a clear solution was obtained. The samples were then cooled to room temperature and distilled (FOSS^TM^ Kjeltec^TM^ 2,200), after which the bluish green distillate was titrated with 0.1 M HCl solution. The acid was added until the first color change was observed. The volume of acid used for titrating each sample and the blank was recorded. The percentage nitrogen was estimated using the formula:

$$\text{\textbackslash{}\% Total nitrogen =}\;\frac{100\times (\text{VA - VB)}\times \text{NA}\times 14}{W\times 1000}$$

where: VA is the volume in ml of standard acid used in titration, VB is the volume in ml of standard acid used in blank, NA is the normality of acid (HCl), and W is the weight in grams of the sample.

#### 2.2.4 Determination of crude fat

The Soxhlet extraction method was used for crude fat determination. In the experiment, 2 g of dried samples from moisture determination was transferred into extraction thimbles and covered with balls of cotton wool. The thimble and its content were placed in a Soxhlet extractor and connected to a weighed dry 500‐ml round bottom flask containing 250 ml of petroleum ether. The apparatus was then connected to a quick fit condenser and refluxed for 16 hr 30 min on an electrothermal extraction unit. The flask was removed after extraction was completed and the petroleum ether evaporated on a rotary water bath (Buchi Rotavapor R‐200). After the evaporation, the flask with the fat was heated in an oven for 1 hr at 103°C, cooled in a desiccator, and weighed. The crude fat content was calculated according to the formula below:

$$\frac{\text{Weight of flask and fat - Weight of flask}}{2}\times 100\%$$

#### 2.2.5 Determination of crude fiber

The defatted sample from the crude fat determination was transferred into a 500‐ml round bottom flask, and 200 ml of boiling 1.25% H_2_SO_4 _was added and then connected to a condenser. The sample was boiled for 30 min after which the content of the flask was filtered. The residue was washed with boiling water until it was no longer acid, and boiled again for 30 min with 200 ml 1.25% NaOH, and filtration was repeated until the residue had no base. The residue was transferred to a Gooch crucible and the remaining particles washed into the crucible with 15 ml 10% HCl and filtered. The crucible and its content were weighed and dried overnight at 105°C, cooled in a desiccator, and reweighed. This was then ashed in a muffle furnace (Thermolyne/Thermo Scientific) at 550°C for 3 hr. The crucible was cooled in a desiccator and reweighed. The difference in the weight of crucible and its content before and after ashing was expressed as the percentage crude fiber.

$$\text{The formula used for crude fiber determination}\frac{\left(X-Y\right)}{W}\times 100$$

where:

X is the weight of crucible and dried sample before ashing, Y is the weight of the crucible and sample after ashing, W is the weight of the sample used in the fat determination.

#### 2.2.6 Determination of carbohydrate and energy

The carbohydrate composition of the biscuits was determined by difference (100‐the sum of the other five determinations. The energy content of the biscuits was also calculated by Atwater's method (AOAC, 1990).

### 2.3 Determination of mineral content

Mineral analysis was done to test for the content of iron, calcium, and zinc on extracted dry ashed samples using an atomic absorption spectrophotometer according to the AOAC (2000) method. Briefly, 5 g of each sample was weighed into a crucible and ashed overnight in a muffle furnace (Thermolyne/Thermo Scientific) at 550°C. The resulting ash was dissolved in 25 ml of 20% HCl at low temperature on a hot plate. The solution was filtered into a 50‐ml volumetric flask, diluted to the volume with deionized water, and mixed thoroughly. For calcium determination, 2.5 ml of Lanthanum chloride solution (1%, w/v) was added to the analyte. All samples were analyzed in duplicate.

### 2.4 Analysis of fatty acid composition

Fatty acid methyl esters (FAMEs) of the biscuit samples were prepared according to the method described by Frank et al. (2006). For each sample, 2 mg of oil was weighed into a 15‐mL capped centrifuge tube, dissolved in 1.5 ml of sodium hydroxide (2M NaOH) in methanol, and heated in a water bath at 100°C for 5 min. After cooling, 12% BF_3_ in methanol was added and the samples were heated again at 100°C for 30 min after 20 ml of hexane was added and stirred. To enhance phase separation, 5 ml of saturated NaCl was added. Supernatants were transferred to a 2 ml autosampler vial and analyzed using gas chromatography (Agilent 6,890, Wilmington, DE, USA). FAME standards were used to determine each type of fatty acid by comparing retention times of fatty acids of analyzed samples with that of reference standards. Results are reported as g fatty acid/100 g of total fatty acids. All samples were analyzed in duplicate.

### 2.5 Microbial safety

The biscuit samples were transferred into sterile sample bags and transported on ice to the laboratory prior to analysis. The samples were stored refrigerated at 5°C for 48 hr after analysis in the Microbial Biotechnology Laboratory of the Department of Biochemistry, KNUST.

#### 2.5.1 Chemical reagents

The agars used were products of OXOID Laboratories, Basingstoke Hampshire, England. They included plate count agar used for the isolation of total viable count, Mannitol salt agar for isolation of *Staphylococcus*, MacConkey agar for total coliform count, and malt extract agar for the isolation and enumeration of fungi.

#### 2.5.2 Preparation of agar

Plate count agar (nutrient agar), MacConkey agar, and Mannitol salt agar were prepared according to standard procedures.

#### 2.5.3 Serial dilution

Serial dilution was done to reduce a dense culture of cells to a more usable concentration. Each dilution done reduces the concentration by a certain amount. A mass of 5 g of each sample was weighed and placed in 45 ml of peptone water solution. Serial dilution was performed for 10^‐1^, 10^−2^, 10^−3^, 10^−4^, 10^−5^, and 10^−6^. The serial dilution done for 10^−3 ^provided very clear and distinct isolate; hence, it was selected for the subsequent analysis (Fung, 1985).

#### 2.5.4 Culturing and isolation of microorganisms

Spread plate technique was used to inoculate the microorganisms. An inoculum volume of 0.1 ml from each dilution series was pipetted to their respective labeled Petri dishes of plate count agar plates. The glass spreader (hockey stick) was sterilized using ethanol and spread over a Bunsen burner. The solution was spread evenly over the agar plate using the hockey stick while carefully rotating the Petri dishes underneath an angle of 45^0^. The Petri dish now containing the agar and evenly distributed sample solution was incubated at 37°C for 24 hr. Different isolates that grew in the agar were classified based on their morphological characteristics. The morphological parameters considered were the shape/form, color, margin, elevation, and optical characteristics of the isolates (Fung, 1985).

#### 2.5.5 Determination of *Staphylococcus aureus*

*Staphylococcus species* were isolated and enumerated by spread plate method and grown on salt Mannitol agar (SMA). Serial dilutions of 10^‐1^ to 10^‐4 ^were prepared by diluting 10 g of sample into 90 ml of sterilized peptone water for stock dilution. One milliliter of aliquots from each of the dilution was inoculated into Petri dishes with already prepared SMA. The inoculum was evenly spread with a sterile bent rod and allowed to dry for 15 min at room temperature. The plates were inverted and incubated at 35 ºC for 24 hr. After incubation, yellow colonies were counted and recorded as *Staphylococcus* counts.

#### 2.5.6 Determination of total coliform count

The TCC was carried out by spread plate method on MacConkey agar (MA). Serial dilutions of 10^‐1^ to 10^‐6 ^were prepared by diluting 10 g of sample into 90 ml of sterilized peptone water for the stock dilution. One milliliter aliquot from each of the dilution was inoculated into Petri dishes with already prepared SMA. The inoculum was evenly spread with a sterile bent rod and allowed to dry for 15 min at room temperature. The plates were inverted and incubated at 35°C for 24 hr.

### 2.6 Sensory evaluation

An acceptability study was done among 130 pregnant women with the biscuit formulation containing 35% PWLF. This formulation alone was used for the acceptability because it had the highest score for texture, aroma, taste, color, and overall acceptability for a descriptive evaluation conducted by 20 semitrained students of the Department of Food Science and technology, KNUST (unpublished data). The evaluation of the biscuit among the pregnant women was based on the following parameters: color, sweetness, aroma, texture, and overall acceptability on a 5‐point hedonic scale with 1 representing the least score (dislike very much) and 5 the highest score (like very much).

### 2.7 Statistical analysis

Results were presented as means and standard deviation. Analysis of variance and Tukey's honestly significant difference (HSD) tests were used to determine the differences among means using SPSS (V.23; IBM SPSS Statistics) at 95% of significance. All experiments were done in triplicates unless stated otherwise.

## 3 RESULTS AND DISCUSSION

### 3.1 Nutritional composition

The nutrient composition of the biscuits enriched with PWL and OFSP is presented in Table 1. The moisture content of the biscuits ranged from 4.0% to 7.07% decreasing with increasing percentage of PWLF substitution. Biscuits produced from 70% PWLF had the lowest (4.0%) moisture content while the biscuits produced from 100% wheat flour had the highest (7.07%). The shelf life of baked products has direct associations with their moisture content, which is an index of water activity and a measure of stability and susceptibility to microbial contamination. Low moisture content of biscuits between 1% and 5% renders them less perishable, thus suggesting that the biscuits produced from 70% PWLF substitution could be stored for a longer time if packaged and stored in appropriate conditions. The carbohydrate content of the biscuits decreased with an increased proportion of PWLF addition. This was expected as increasing PWLF substitution reduced the amount of wheat flour used. The protein content of the biscuits ranged from 8.01 g to 11.68 g. A progressive increase in the protein content in proportion to the percentage of PWLF added was observed. Previous studies have reported that *Rhychophorus Phoenicis* is a good source of digestible protein, with a protein content of 25% (dry matter) (Okunowo, Olagboye, Afolabi, & Oyedeji, 2017), and this perceptibly is the reason for the biscuits with 70% PWLF substitution recording a higher protein content as compared with the 100% wheat flour.

**Table 1:** Proximate and mineral composition of biscuits per 100 g

Nutrient	PB	RA	DA
Protein	8.01 ± 0.05^a^	9.63 ± 0.08^b^	11.68 ± 0.42^c^
Fat	23 ± 0.5^a^	34±2^b^	44.33 ± 2.08^c^
Ash	1.61 ± 0.01^a^	1.64 ± 0.01^b^	2.10 ± 0.01^c^
Moisture	7.07 ± 0.51^a^	7.67 ± 0.58^a^	4.0 ± 0.5^b^
Fiber	3 ± 0^a^	4 ± 0^a^	5 ± 1^b^
Carbohydrate	57.49 ± 1.29^a^	43.07 ± 2.6^b^	32.88 ± 1.86^c^
Energy	468.97 ± 0.65^a^	516 ± 7.83^b^	577.12 ± 15.15^c^
Calcium	29.81 ± 0.52^a^	31.56 ± 1.2^a^	41.29 ± 1.37^b^
Iron	9.03 ± 0.17^a^	9.12 ± 0.29^a^	10.12 ± 0.19^c^
Zinc	0.4±0^a^	1.26±0^b^	2.47 ± 0.06^c^

Note

Data presented are proximate and mineral compositions of developed biscuits. PB—plain wheat biscuits (100% wheat), RA—regular PWL biscuits (35% PWL, 15% OFSP), and DA—double PWL biscuits (70% PWL, 15% OFSP). Means with different superscripts (alphabets) are significantly different, *p* < 0.05

The protein content of the biscuits developed in this study is lower than those obtained by Adeboye, Bolaji, and Fatola (2016) and Idolo (2010) in PWLF‐enriched cookies and wheat burns, respectively. It is important to note, however, that the formulations used by Adeboye et al. (2016) and Idolo (2010) had an additional source of protein being eggs, but this study did not. The difference in protein content could therefore be attributed to this factor. Again, since the nutrient composition of edible insect is influenced by their feed; another reason for the difference in protein content could be that the protein levels of PWL used in the formulation in this study were generally lower than the ones used by Adeboye et al. (2016) and Idolo (2010). Furthermore, defatted palm weevil larva (dry basis) has been found to have a protein content of 66.3% (Elemo et al., 2011). This implies that the protein content of the biscuit developed in this study can be improved by defatting of the larvae used in the production of PWLF. The results of the study indicate that the protein content of the biscuits developed can meet about 16% of the dietary reference intake of protein for pregnant women, which is 71 g, making it a good addition to the diet of pregnant women. The protein quality of food is dependent on its essential amino acid content. An amino acid profile evaluation of palm weevil larvae revealed that it contained isoleucine, leucine, lysine, methionine, histidine, phenylalanine, threonine, tryptophan, and valine which are all essential amino acids. Leucine and histidine are known for enhanced growth in infants and young children making the biscuits suitable for infants and young children as well. Also, adding palm weevil larvae flour to wheat flour in snack production would provide the lysine and threonine lacking in the wheat. This could contribute to the reduction of protein‐related malnutrition cases in many developing countries like Ghana where starchy foods are major staples.

The fat, fiber, and energy levels increased as PWLF substitution increased. This does not pose a problem because pregnant women need extra calories to support the development of the growing fetus. Palm weevil larvae are known to contain 65% fat, predominantly composed of saturated fats. Caproic acid and arachidic acid were the most abundant fatty acids in all three formulations, contributing between 43.3% and 50% and 36% and 43.5% of total fatty acids, respectively (Table 2). The biscuits also contained appreciable amounts of caprylic acid (4.8% to 5.5% and docosahexaenoic acid (4.3% to 7.3%). The fats were predominantly saturated (92.5% to 95.5%). Several studies have reported a number of health benefits for medium‐chain fatty acids (MCFAs) including caproic acid. It was found that consumption of MCFAs resulted in a reduction in fat deposition through enhancement of thermogenesis in rats (Papamandjaris, MacDougall, & Jones, 1998). Clinical studies in both healthy and obese human subjects found greater fat oxidation and postprandial energy expenditure after consumption of medium‐chain fats (St‐Onge, Peter, & Jones, 2002).

**Table 2:** Fatty acid profile of biscuits (g/100 g)

Fatty Acid	PB	RA	DA
C6:0	43.53 ± 7.30^a^	50.14 ± 0.39^a^	43.96 ± 1.14^a^
C8:0	5.53 ± 0.33^a^	6.81 ± 0.49^ab^	4.85 ± 0.42^ac^
C10:0	tr	tr	0.85 ± 0
C12:0	tr	tr	tr
C14:0	0.14 ± 0.03	tr	tr
C16:0	0.87 ± 0.12^a^	0.50 ± 0.01^bd^	0.44 ± 0.01^cd^
C18:0	1.3 ± 0.17^a^	1.57 ± 0.04^ad^	2.33 ± 0.01^ce^
C18:1n9	tr	tr	tr
C18:2n6	tr	0.10 ± 0.07	tr
C20:0	40.07 ± 5.58^a^	36.12 ± 0.83^a^	43.49 ± 0.1^a^
C22:6n3	7.31 ± 0.96^a^	4.64 ± 0.09^bd^	4.36 ± 0.1^cd^
SFA	92.56 ± 0.97^a^	95.22±0^bd^	95.55 ± 0.01^cd^
MUFA	tr	tr	tr
PUFA	7.34 ± 0.97^a^	4.74 ± 0.02^bd^	4.42 ± 0.01^cd^

Note

Data are expressed as means ± standard deviation (*n* = 2). SFA—saturated fatty acids; MUFA—monounsaturated fatty acids; PUFA—polyunsaturated fatty acids. tr represents trace amount (60.1 g/100 g fatty acids). PB–plain wheat biscuits (100% wheat), RA—regular PWL biscuits (35% PWL, 15% OFSP), and DA—double PWL biscuits (70% PWL, 15% OFSP). Means with different superscripts (alphabets) are significantly different, *p* < 0.05.

The ash, calcium, iron, and zinc content of the biscuits also followed the trend of progressive increase with the highest values observed for the 70% PWLF substitution. The high ash, zinc, calcium, and iron contents of the biscuits with the highest PWLF substitution can be attributed to the fact that animal source foods are high in essential micronutrients. Micronutrients are essential for life and for optimal functioning of the body. The formulated product in this study could thus help combat the micronutrient deficiencies which are widespread among pregnant women and children under 5 years globally, Ghana being no exception. Iron, iodine, folate, vitamin A, and zinc deficiencies are the most common and lead to the growth and intellectual impairments, maternal and neonatal complications, and increased risk of morbidity and mortality. According to Ekpo and Onigbinde (2005), the consumption of 100 g of PWL could meet the RDA for iron, zinc, copper, magnesium, and manganese in most developed countries.

### 3.2 Microbial quality of biscuits

It has been reported that insects, in general, can harbor *Salmonella *Spp.,* Campylobacter,* and *Staphylococcus *Spp. and fluke foodborne and waterborne pathogens and chemical hazards (Belluco et al., 2013), and this has raised a lot of concerns about the utilization of edible insects as food. Results of the microbial quality test of the biscuits showed that they are safe for consumption (Table 3).

**Table 3:** Microbial quality of formulated biscuits

Sample ID	TAC (cfu/g)	TCC (cfu/g)	*Staphylococcus aureus*	Salmonella	Fungi
PB	<30	0.00	None detected	None detected	0.00
DA	3.8×10^3^ ±3.61	0.00	None detected	None detected	0.00
RA	<30	0.00	None detected	None detected	0.00

Note

Data presented are microbial content of developed biscuits. CFU—count‐forming units; TAC—total aerobic counts; TCC—thermotolerant coliform counts. PB—plain wheat biscuits (100% wheat), RA—regular PWL biscuits (35% PWL, 15% OFSP), and DA—double PWL biscuits (70% PWL, 15% OFSP).

### 3.3 Sensory evaluation of biscuits

**Figure 2:**
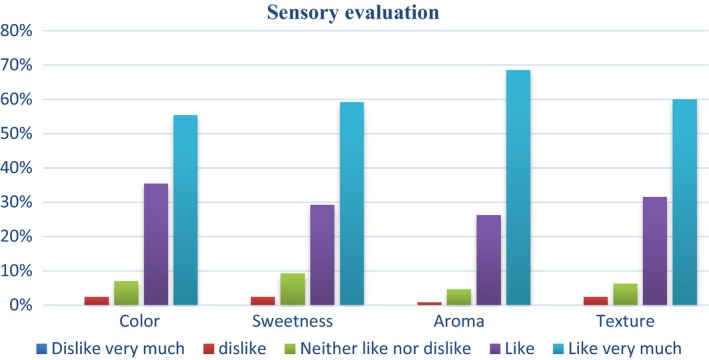
Sensory evaluation of developed biscuits

**Figure 3:**
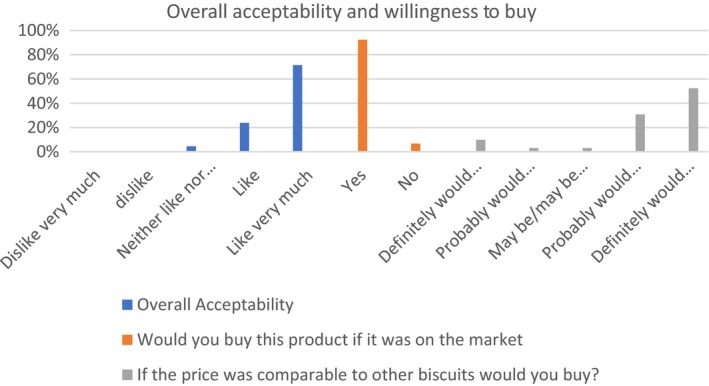
Overall acceptability and willingness to buy

Figure 2 shows the acceptability of the developed biscuits among 130 pregnant Ghanaian women. It was observed that majority of the pregnant women liked the biscuit very much in terms of the color, sweetness, aroma, and texture and are willing to buy the biscuits if it is sold on the market (Figures 2 and 3). The analysis of variance (ANOVA) showed that the overall acceptability of the biscuits was found to be significantly dependent on the sweetness (*p* < 0.05). The acceptability was done among pregnant women because the study aimed to develop a product that would be acceptable among them and can be used to supplement their protein and iron intake to reduce the incidence of anemia and severe acute malnutrition. It seemed from the study that the use of palm weevil larvae in biscuit production reduced the stigma associated with edible insect consumption, thus making it easier for pregnant women to accept the product.

## 4 CONCLUSION

The nutrient composition of the formulated product compared to 100% wheat flour biscuits was higher. The trend observed was that increasing the percentage of PWLF was directly proportional to an increase in nutrient content. Microbial analysis deemed the formulated product safe for consumption. The formulated product was also accepted according to the sensory evaluation of its sweetness, color, aroma, and texture. This study has shown that it is possible to use palm weevil larvae, orange‐fleshed sweet potato, and wheat composite flour to produce acceptable biscuits of high nutritional value.

Further studies can investigate the effects of defatting of the larvae on the protein and micronutrient composition of the biscuits and the bioavailability of the nutrients as well as the impact of the biscuits on the nutritional status of pregnant women to provide adequate evidence for promoting edible insect, specifically palm weevil larvae and orange‐fleshed sweet potato consumption.

## CONFLICT OF INTEREST

The authors declare no conflict of interest to disclose.

## ETHICAL CONSIDERATION

The study was conducted in accordance with the Helsinki guidelines for human subjects. The protocol and procedures for the sensory evaluation were reviewed and approved by the Committee for Human Research and Publication Ethics (CHRPE/KNUST) of the Kwame Nkrumah University of Science and technology. Written permission to carry out the research was obtained from the medical superintendents of the hospitals from where pregnant women were contacted. Verbal and nonverbal consent was also sought from each pregnant woman for voluntary participation.
